# High Precision Detection Pipe Bursts Based on Small Sample Diagnostic Method

**DOI:** 10.3390/s25113431

**Published:** 2025-05-29

**Authors:** Guoxin Shi, Xianpeng Wang, Jingjing Zhang, Xinlei Gao

**Affiliations:** 1 School of Information and Communication Engineering, Hainan University, Haikou 570228, China; hainansgx@126.com; 2 Guangdong Water Co., Ltd., Shenzhen 518021, China; gaoxinleiuser@126.com

**Keywords:** pipe bursts detection, deep transfer learning, small sample diagnostic, high precision

## Abstract

In order to improve the accuracy of pipe burst detection in water distribution networks (WDNs), a novel small sample diagnosis method (SSDM) based on the head loss ratio (HLR) method and deep transfer learning (DTL) method has been proposed. In this paper, the burst state was quickly detected through the limited data analysis of pressure monitoring points. The HLR method was introduced to enhance data features. DTL was introduced to improve the accuracy of small sample burst detection. The simulated data and real data were enhanced by HLR. Then, the model was trained and obtained through the DTL. The performance of the model was evaluated in both simulated and real scenarios. The results indicate that the leaked features can be improved by 350% by the HLR. The accuracy of SSDM reaches 99.56%. The SSDM has been successfully applied to the detection of real WDNs. The proposed method provides potential application value for detecting pipe bursts.

## 1. Introduction

Pipe bursts in the water distribution networks (WDNs) lead to the risk of bacteria and contaminants entering the interior of the pipes, in addition to wasting large amounts of water and causing serious damage to other nearby facilities [1]. Statistics show that leaks reach 20–30% or more of the total water supply in distribution networks in various countries [2]. Water loss in China reached about 78.5 billion cubic meters in 2017 [3], where pipe bursts in WDNs are a main source of water loss. Therefore, it is crucial to diagnose pipe bursts in WDNs in a timely and accurate manner. Conventional detection methods of WDNs leakage/burst include experienced methods, portable equipment methods, sensor-based methods, and model-based methods [4,5,6]. Experience-based methods mainly rely on human experience and masters with specialized technology to observe whether there is leakage or not [7]. Portable equipment methods include ground penetrating radar [8], listening stick [9], and the remote field eddy current (RFEC) technique [10]. Sensor-based methods can be divided into acoustic sensor methods, infrared thermography methods [11] and distributed fiber optic sensor methods [12,13]. The model-based approach constructs a mathematical model based on the topology of the WDNs. The leakage detection is determined by comparing the estimated value of the pipe network with the real value according to the model [14,15]. Although traditional methods are effective in detecting leakage in WDNs, these methods are inefficient and costly, and it is difficult to achieve the real-time monitoring of large-scale WDNs. Mazzoni et al. proposed an automated pressure-based method for detecting and pre-locating the anomalous consumption events in distribution networks by analyzing the trend of differential pressure over time to detect and pre-locate the time of anomalous water consumption [16]. Therefore, it is crucial to investigate the efficient detection of WDN burst detection methods.

With the advancements in the big data and supervisory control and data acquisition (SCADA) system, data-driven WDNs burst detection methods are rapidly developing [17]. The real-time monitoring data from SCADA systems have provided effective support for leak detection in urban water networks, which has led to the emergence of a number of methods that utilize real-time monitoring data to determine leakage. Artificial intelligence methods are utilized in data-driven burst detection. The core idea is to extract the features of a large amount of labeled leakage and non-leakage data. Aksela et al. used the self-organization map (SOM) to classify the pressure and flow data, hence obtaining high accuracy [18]. Mounce et al. used support vector regression (SVR) to obtain the classification results [19]. Mounce and Machell used an artificial neural network (ANN) for the classification of time series, and the results showed that ANN could be effectively used for leak detection tasks [20]. Zhou et al. constructed a fully linearly connected neural network for leak detection in pipeline networks [21]. Although these machine learning methods have been used to detect leakage emergencies in recent years, they require a large amount of training data. Compared to tasks in computer vision and audio processing refs. [22,23], obtaining accurate leakage data from pipelines requires significant manual and resource consumption. When the available labeled training data are limited, intelligent fault diagnosis models may encounter problems such as overfitting and the inability to recognize distinguishing features. Therefore, it is important to study small-sample diagnostic methods for detecting leaks in WDNs.

The research on small-sample intelligent fault diagnosis methods is currently in full swing. Wang et al. proposed a one-stage self-supervised momentum contrastive learning for small-sample open set fault diagnosis [24]. Ding et al. applied MoCo to extract local representations from unlabeled running to fault vibration signals, effectively capturing the early fault features of rolling bearings [25]. Anass et al. proposed an encoder–decoder self-supervised learning framework to address the data scarcity problem in prediction [26]. Li et al. studied the open set recognition (OSR) fault diagnosis of nuclear power plants (NPP) and proposed a novel OSR NPP fault diagnosis framework based on convolutional prototype learning (CPL) and prototype matching by distance (PMD) [27].

DTL is also one of the effective methods for solving intelligent fault diagnosis problems. Transfer learning solves problems in new domains by utilizing features common to two or more different but related domains [28,29,30]. For example, Mao et al. designed a deep domain adaptive neural network for the early fault detection of rolling bearings, effectively extracting domain invariant feature representations [31]. Yang et al. proposed a novel mechanical fault detection framework based on contrastive representation, which uses contrastive learning to extract the intrinsic representations of health status data [32]. Wang Chuang et al. used contrastive adversarial networks to diagnose pipeline faults under small sample conditions [33]. Note that most current methods rely on building negative sample pairs to learn latent representations, and many methods are based on expensive high-frequency sensor data, refs. [31,32] make it available only for high-frequency fault diagnosis.

In this paper, a novel small-sample diagnosis method (SSDM) based on the head loss ratio (HLR) method and deep transfer learning (DTL) method has been proposed. The HLR was introduced into neural networks to enhance data features. As a physics-guided feature enhancer, HLR derived from hydraulic equations is embedded into neural network layers to compensate for sparse data distributions. This transforms raw pressure signals into domain-invariant head loss patterns, effectively amplifying leakage-induced hydraulic transients. DTL was introduced to improve the accuracy of small sample burst detection. A domain adaptation mechanism bridges simulated (source domain) and real-world (target domain) data. Through the minimization of cross-correlation distance, the model prevents negative transfer while extracting transferable leakage feature across domains. The main contributions of this article are as follows:

1. HLR is embedded as a physical paradigm in the hybrid modeling of deep transfer learning;
2. A novel feature extraction mechanism for small-sample domain adaptation pipelines;
3. The proposed SSDM framework does not rely on high-frequency pressure sensors (high frequency means sampling frequency higher than 100 HZ). The deployment of low-frequency pressure sensors greatly reduces the engineering budget and has strong practical value.

## 2. Methodology

### 2.1. Overview

**Figure 1:**
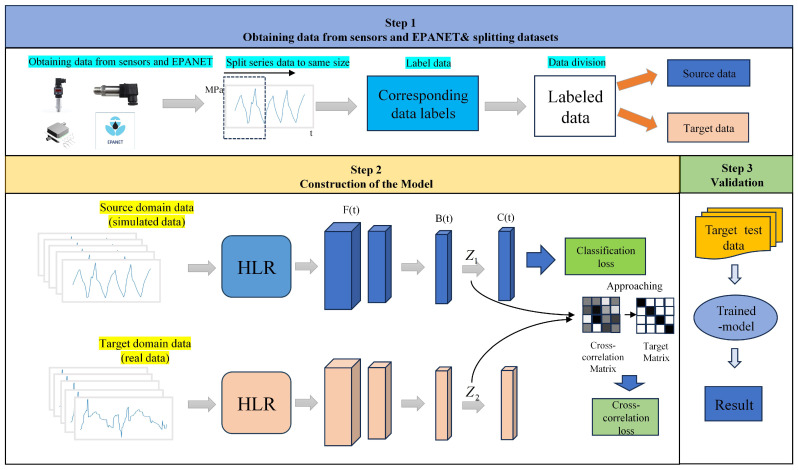
A visualization of the proposed framework.

Figure 1 shows a visualization of the proposed small-sample diagnostic method (SSDM) based on deep neural networks. The proposed SSDM is constructed based on a hydraulic model water distribution networks (WDNs). Hydraulic features are extracted from each pipe when WDN burst occurred. Based on the fact that burst pipes cause pressure fluctuations around the burst point, the WDN leaks are detected by learning the pressure characteristics of bursts at different locations. The target domain data referred to in Figure 1 are real-world pressure data. However, due to the limited availability of real-world data and in order to analyze more different situations, in case 1, the data are simulated, and in case 2, the target domain data are real-world data.

**Figure 2:**
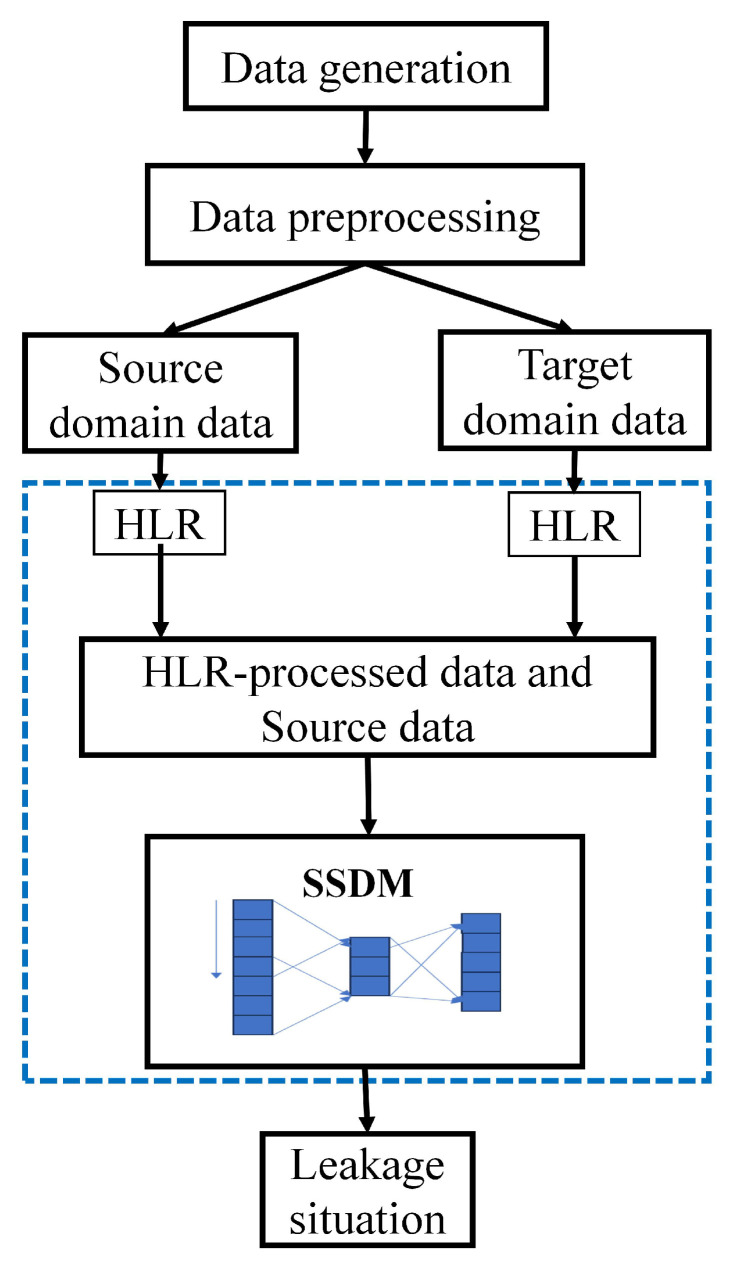
Flowchart of the SSDM.

Figure 2 shows the flowchart of the SSDM detection process, as follows:

Data were collected and processed. Experiments on simulated leakage were conducted using Environmental Protection Agency Network Analysis Tool (EPANET). Leakage and non-leakage data from different nodes were simulated separately under pressure-driven analysis (PDA) to obtain pressure patterns at different points or targets in the network. The detailed steps of data generation and processing were described in Section 2.2 and Section 2.3.

A small-sample diagnosis method based on neural networks was proposed. The model is constructed in Section 2.4. The data were subjected to operations such as feature extraction classification and gradient backpropagation so that the neural network learned a robust representation of the leakage.

### 2.2. Data Generation

EPANET was used to construct a hydraulic model of the WDN and perform simulations in this study. Demand-driven (DDA) hydraulic model-based and pressure-driven (PDA) hydraulic model-based were two simulation scenarios in EPANET simulation. In this paper, the model simulation was based on PDA. The pressure demand at each node of the pressure driven based model was as follows:

(1) $$q_{i}^{*}=\left\{\begin{array}{cc} 0, & H_{i}^{*}<H_{i}^{\min}\\ q_{i}^{req}{\left(\frac{H_{i}^{*}-H_{i}^{\min}}{H_{i}^{req}-H_{i}^{\min}}\right)}^{\frac{1}{\eta }}, & H_{i}^{\min}<H_{i}^{*}<H_{i}^{req}\\ q_{i}^{req}, & H_{i}^{*}>H_{i}^{req} \end{array}\right.$$

where $q_{i}^{*}$ is the available outflow flow rate of the node, $q_{i}^{req}$ is the demand flow rate of the node, $H_{i}^{\min}$ is the minimum pressure demanded by the node, $H_{i}^{req}$ is the demand pressure magnitude of the node, and $H_{i}^{*}$ is the actual pressure magnitude of the node.

The simulation of leaks was performed by adding burst in EPANET. When a pipe bursts, the flow rate of the burst outflow was determined by a combination of factors, which was given by

(2) $$q_{burst}=CA_{o}\sqrt{2gH_{o}}$$

where $q_{\mathrm{burst}}$ is the pipe burst for the flow rate of the outflow, *C* is the outflow coefficient, C=0.5∼0.7 depends on the Reynolds number and the shape of the orifice. In this paper, we only simulated leaks at different locations and with different radii, without changing the shape of the aperture. Therefore, the value of *C* was not varied and the same value as in the literature was taken, and is set as 0.6 in this paper [21]. $A_{o}$ is the area of the orifice, which is determined by the type, thickness, and diameter of the material. *g* is the gravity coefficient and $H_{o}$ is the pressure.

We can employ EPANET to simulate the burst (orifice outflow) on pipes, and $A_{o}$ is the only parameter to be determined. For a WDN, the material thickness of each pipe is determined, so we only need to determine the diameter of the leak orifices to simulate the different sizes of leaks.

In order to simulate different cases of leakage and try to cover bursts of different sizes and leak locations, the parameters were varied in the following ways:

1. Burst hole size. Different burst hole size could cause different pressure fluctuations. If the burst hole size was too small, such as 0.01 mm, leakage may not be detected because of too small pressure fluctuations;
2. The location of the burst. When a leak occurred at a different location, the information captured by the different pressure sensors could vary;
3. Duration of leaks. According to the literature [21], the node pressures exhibited different pressure characteristics driven by water used behavior at different times of the day. The longer the duration, the higher the prediction accuracy (prediction accuracy refers to the accuracy of classifier prediction);
4. Sensor sampling interval. The accuracy and resolution of the data was determined by the sampling interval of the sensor. More subtle data features were obtained with a higher sampling frequency, but that also means that a larger amount of data were required. A suitable sampling frequency should be selected in combination with hardware realizability and reality.

### 2.3. Data Enhancement and Preprocessing

The form of one pressure sensor data can be expressed as follows:

(3) $$P_{b}={\left[P_{1b},P_{2b},\ldots ,P_{ab}\right]}^{\prime }$$

where *a* denotes the length of the pressure data, which is calculated as $a=\frac{T_{d}}{\delta }$, $T_{d}$ denotes the duration, and δ denotes the sensor sampling interval. Thus, the data form of a simulation is as follows, with *b* denoting the number of pressure sensors:

(4) $$P=\left[P_{1},P_{2},\ldots ,P_{b}\right]=\left[\begin{array}{ccc} P_{11} & \ldots & P_{1b}\\ \vdots & \ddots & \vdots \\ P_{a1} & \ldots & P_{ab} \end{array}\right]$$

To make the simulated data close to the real data and prevent data overfitting, an additive Gaussian noise with a mean of 0 and variance of $\sigma^{2}$ and a random perturbation of (−10, 10) were added to the source domain data [34].

#### Head Loss Ratio

For the given time *t*, HLR for a triple of different pressure measurements $p_{1}\left(t\right),p_{2}\left(t\right),p_{3}\left(t\right)$ is given by

(5) $$HLR\left(p_{1},p_{2},p_{3},t\right)=\frac{p_{1}\left(t\right)-p_{2}\left(t\right)}{p_{2}\left(t\right)-p_{3}\left(t\right)}$$

if $p_{2}\left(t\right)-p_{3}\left(t\right)=0$, then $HLR(p_{1},p_{2},p_{3}):=\infty$.

In Formula (5), the $p_{1}\left(t\right)-p_{2}\left(t\right)$ reflects the head loss in the upstream section, and the $p_{2}\left(t\right)-p_{3}\left(t\right)$ reflects the head loss in the downstream section. The HLR amplifies the local pressure gradient changes caused by leakage through ratio amplification. When a leak occurs, the downstream pressure drops sharply, resulting in a decrease in the denominator and a significant increase in the HLR.

The initial pressure datum *P* is processed via Equation (5) by iteratively selecting three distinct connected nodes, yielding data formatted as:

(6) $$P_{F}=\left[P_{1},P_{2},\ldots ,P_{b},P_{HLR}\right]$$

Assuming that all pressure monitoring points in WDN are connected, then $P_{HLR}$ is the data in row *a* and column $C_{b}^{3}$.

The proposed pressure datum $P_{F}$ is adopted as the training data. To validate its effectiveness, we provide the following theoretical analysis:

For a given WDN and time span, assume the following Assumptions [35]:

1. The WDN has only one water source.
2. The WDN has no leak inside.
3. The WDN has no pump working inside.
4. There exists a function $f\left(t\right):R_{+}\to R$, for any water consumption $d_{i}\left(t\right)$ at node *i* in the WDN, which satisfies:

(7) $$d_{i}\left(t\right)=a_{i}f\left(i\right)$$

where $a_{i}$ represents non-negative constant.

Then, all HLRs in the given WDN should be constant for all $t\in [t_{1},t_{2}]$.

This operational assumption implies that, in a WDN with uniform consumption patterns among users, the observed HLR deviation from normal operating values under single-reservoir operation with all pumps deactivated serves as a leakage indicator. Specifically, when the network operates in this simplified configuration (a single active reservoir, pumps offline, and consistent demand patterns), hydraulic anomalies manifested through HLR deviations from baseline values reveal pressure inconsistencies characteristic of leakage scenarios. The constructed dataset $P_{F}$ provides the neural network with distinct leakage/non-leakage features through these measurable hydraulic responses. For non-leak conditions, the predicted HLR ($P_{HLR}$) maintains temporal stability at monitoring time of t=T, whereas leakage conditions induce measurable $P_{HLR}$ variations over time due to progressive pressure losses.

### 2.4. Placement of Pressure Monitoring Points

As an important prelude step to leak detection, the effectiveness of leak detection is directly influenced by pressure sensor placement. In the actual project, it is unrealistic to arrange pressure monitoring points at each node in the WDNs. If the pressure monitoring point is not properly placed, the limited pressure monitoring points could not be able to detect the leakage fluctuations at any location in the WDNs. In this paper, we chose the K-means algorithm to place the row vector. The clustering center is the point to realize the arrangement of pressure monitoring points. The pressure sensitive matrix can reflect the degree of influence of the flow change of the node on the pressure fluctuation of the surrounding nodes. Its calculation formula is as follows:

(8) $$\frac{\partial Q}{\partial H}=-{\left(ABA^{T}\right)}^{-1}$$

where *A* is the incidence matrix describing the network topology [36], and the elements in matrix *A* can be determined as

(9) $$A\left(i,j\right)=\left\{\begin{array}{cc} -1 & \mathrm{if}\;\mathrm{node}\;i\;\mathrm{is}\;\mathrm{the}\;\mathrm{initial}\;\mathrm{node}\;\mathrm{to}\;\mathrm{element}\;j\\ 0 & \mathrm{if}\;\mathrm{node}\;i\;\mathrm{is}\;\mathrm{not}\;\mathrm{connected}\;\mathrm{to}\;\mathrm{element}\;j\\ +1 & \mathrm{if}\;\mathrm{node}\;i\;\mathrm{is}\;\mathrm{the}\;\mathrm{final}\;\mathrm{node}\;\mathrm{of}\;\mathrm{element}\;j \end{array}\right.$$

(10) $$B=\left[\begin{array}{cccc} 1.852\frac{q_{1}}{h_{1}} & 0 & \ldots & 0\\ 0 & 1.852\frac{q_{2}}{h_{2}} & \ldots & 0\\ \vdots & \vdots & \ddots & \vdots \\ 0 & 0 & \ldots & 1.852\frac{q_{m}}{h_{m}} \end{array}\right]$$

where $q_{m}$ is the flow rate of pipe *m* and $h_{m}$ is the pressure loss in pipe *m*.

The idea of K-means algorithm is based on the similarity measure. Similar samples are grouped into one subset, the similarity of samples in the same subset is minimized and the similarity of different subsets is maximized. First, nodes are randomly selected as initial clustering centers. By minimizing the objective function in Equation (11), the clustering center is continuously updated. Until the objective function (11) is smaller than the specified threshold or reaches the specified number of cycles, ending the cycle. The final clustering result is obtained.

(11) $$J=\min\left\{\sum_{n=1}^{N}\sum_{k=1}^{K}r_{nk}{∥x_{n}-\mu_{k}∥}^{2}\right\}$$

### 2.5. Transfer Learning

The structural diagram of the network is shown in the second step of Figure 1. The proposed SSDM consisted several key components: the feature layer F(t), the bottleneck layer B(t), the classification layer C(t), and the loss function *L*.

The source domain data and the target domain data were extracted from the high-dimensional features through the feature layer. And, the high-dimensional features were mapped to the low-dimensional space through the bottleneck layer, respectively. The source domain feature representation was utilized in this framework to predict the target domain feature representation. The domain shift was reduced by domain adaptation. Finally, the classification results were obtained based on the classification layer. The weights and biases were updated based on the newly constructed loss function *L*.

**Figure 3:**
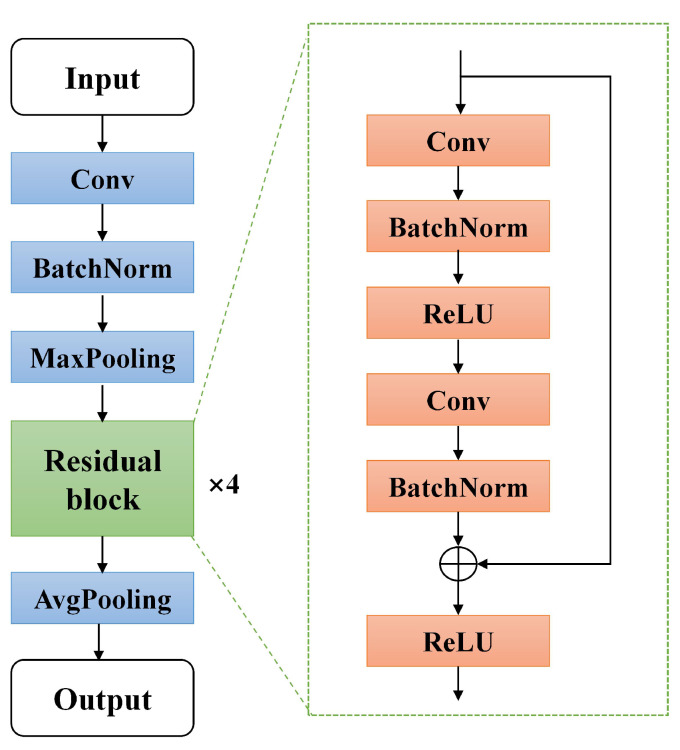
The structure of ResNet-18.

- Feature layer: The feature layer consisted of the classical Resnet18 (no classification layer) [37]. The structure is shown in Figure 3. The structure consisted of a convolutional layer, pooling layer, and residual module. The front three of these layers were the convolutional neural network (CNN). The four residual modules with the same structure were connected after the batch norm layer. The four structures consisted of shortcut connections in the residual module. The gradient explosion problem of the network was avoided by residual connections which improved the stability of the network. Data *x* were fed into feature layer F(t) to produce F(x), where F(t) denotes the function fitted by the feature layer;
- Bottleneck layer: The bottleneck layer was designed to transform the high dimensional features into low dimensional features. The source domain data features was denoted as $Z_{1}=B\left(F\left(x\right)\right)$ and the target domain data features was denoted as $Z_{2}=B\left(F\left(x\right)\right)$ denotes, where B(t) denotes the function fitted by the bottleneck layer;
- Classification layer: The Classification layer was composed of a fully connected linear neural network. The low dimensional features B(F(x)) were fed into the classification layer to obtain the final output C(Z), where C(t) denotes the function fitted by the bottleneck layer.

Loss: In order to achieve knowledge transfer, this paper innovatively proposed a cross-correlation loss to minimize the inter-domain distance. Knowledge transfer in this method refers to the transfer of leakage features and non-leakage features from other pipe networks (pipe networks with a large amount of data) to the target pipe network, thus achieving feature confusion and improving the detection accuracy of the target pipe network classifier. That is, by constructing an inter-domain loss function, thus realizing domain confusion between the source and target domains. The categorical cross-entropy loss and the cross-correlation loss after regularization were used as the back-propagation loss of the network. The classifier has both enough deep features to distinguish the data and is not affected by the differences between the source and target domains. If only the categorical cross-entropy loss was present, it could cause the network to overfit the source domain data, resulting in the target domain data being less accurately classified. If only the cross-correlation loss was minimized, the features were degraded. Source and target domain data were projected onto a single point, resulting in a near-zero cross-correlation loss [38]. Joint training using categorical cross-entropy loss and cross-correlation loss allowed the network to achieve good features. The following was the definition of the loss function:

The total loss function is given by

(12) $$L=L_{CLASS}+\alpha L_{CROSS}$$

where *L* is the total loss function, $L_{CLASS}$ is the categorical cross-entropy loss, α is the regularization term, which is used to weigh the fitness of the source domain and the classification accuracy, and $L_{CROSS}$ is the cross-correlation loss, which is introduced in the following.

Cross-correlation loss measured the correlation between the source and target domains [39]. The prediction of a given sample should be correlated with its corresponding augmented temporal feature, but not with the temporal features of the rest of the samples in the batch dimension. Therefore, the correlation matrix between the source domain predictions and the augmented temporal features should be close to the unit matrix. In other words, the diagonal elements should be close to 1 and the off-diagonal elements should be close to 0. The definition of the cross-correlation loss function was

(13) $$L_{CROSS}=\sum_{i=1}^{N}{\left(1-C_{ii}\right)}^{2}+\lambda \sum_{i=1}^{N}\sum_{j=1,j\neq i}^{N}C_{ij}^{2}$$

where λ is a constant that weights the contribution of the uncorrelated terms. *N* is the dimension of $Z_{1}$ and $Z_{2}$, *i*, and *j* are the indexes of the dimensions, and *C* is the inter-correlation matrix of $Z_{1}$ and $Z_{2}$ along the batch dimensions:

(14) $$C=\frac{1}{N}\frac{Z_{1}^{T}}{{∥Z_{1}∥}_{2}}\frac{Z_{2}}{{∥Z_{2}∥}_{2}}$$

where ${∥\cdot ∥}_{2}$ is the l2 norm, ensuring that $Z_{1}$ and $Z_{2}$ lie in a comparable range.

The cross-correlation loss function was similar to the common transfer learning loss function. However, the cross-correlation loss function differs from the common loss function. The cross-correlation loss function model is efficient and scalable due to the fact that it does not require the construction of a large number of negative samples. By using a cross-correlation loss function, potential representations were learned by the model and then used to distinguish between source and target domain correlations. The algorithm for SSDM is outlined in pseudocode in Algorithm 1.

**Algorithm 1:** SSDM algorithm.**Input:**
Head loss ratio function HLR, feature net F(t), bottleneck B(t), classification net C(t), training epoch *V*, epoch index *v*, batch size *B*, batch index *b*, dataset *D***Output:**
Feature net F(t), bottleneck B(t) and classification net C(t)
1   
Randomly initialize F(t), B(t) and C(t)
2   
**for** 
v∈{1,…,V}
 **do**
3
     **for** b∈{1,…,B} **do**4
    Sample source and target domain data from dataset:                   $x_{s}∼D$,  $x_{t}∼D$5
    Enhance data through the HLR layer:
                  ${\tilde{x}}_{s}\leftarrow HLR\left(x_{s}\right)+x_{s}$

                  ${\tilde{x}}_{t}\leftarrow HLR\left(x_{t}\right)+x_{t}$
6
    Obtain representations:                   $F({\tilde{x}}_{s})$,    $F({\tilde{x}}_{t})$7
    Transform to low dimension:
                   $Z_{1}\leftarrow B\left(F\left({\tilde{x}}_{s}\right)\right)$

                   $Z_{2}\leftarrow B\left(F\left({\tilde{x}}_{t}\right)\right)$
8
    Classification result:
                   $Label\leftarrow C(Z_{1})$
9
    Compute cross-correlation matrix:
                   $C\leftarrow \frac{1}{N}\cdot \frac{Z_{1}^{⊤}}{{∥Z_{1}∥}_{2}}\cdot \frac{Z_{2}}{{∥Z_{2}∥}_{2}}$
10
    Compute loss:
                $L_{\mathrm{CROSS}}\leftarrow \sum_{i=1}^{N}{\left(1-C_{ii}\right)}^{2}+\lambda \sum_{i=1}^{N}\sum_{\begin{array}{c} j=1\\ j\neq i \end{array}}^{N}C_{ij}^{2}$

                   $L\leftarrow L_{\mathrm{CLASS}}+\alpha L_{\mathrm{CROSS}}$
11    
Update F(t), B(t) and C(t)12   **end for**13   
**end for**


### 2.6. Distribution Distance

Prior to model training, it is essential to measure the similarity between the source and target domains to prevent negative transfer caused by significant distribution discrepancies. This study employs two widely adopted distribution distance metrics in domain adaptation/generalization (DA/DG) research: maximum mean discrepancy (MMD) and COR relation alignment (CORAL).

MMD: MMD maps data into a reproducing kernel Hilbert space (RKHS) through kernel functions and computes the distance between domain means in this space. The formulation is given by

(15) $$d_{mmd}\left(h_{s},h_{t}\right)=\frac{1}{n_{s}^{2}}\sum_{i,j=1}^{n_{s}}k\left(h_{si},h_{sj}\right)+\frac{1}{n_{t}^{2}}\sum_{i,j=n_{s}+1}^{n_{s}+n_{t}}k\left(h_{si},h_{sj}\right)-\frac{2}{n_{s}n_{t}}\sum_{j=n_{s}+1}^{n_{s}+n_{t}}k\left(h_{si},h_{sj}\right)$$

where k(·,·) represents the kernel function such as RBF kernel and linear kernel, and $n_{s}=\left|h_{s}\right|$, $n_{t}=\left|h_{t}\right|$ represents the quantity of two distribution data.

CORAL: CORAL quantifies the domain discrepancy by aligning the covariance matrices between the source and target domains:

(16) $$d_{coral}\left(h_{s},h_{t}\right)=\frac{1}{4q^{2}}{∥C_{s}-C_{t}∥}_{F}^{2}$$

where *q* represents the dimension of data, and $C_{s}$ and $C_{t}$ represent the covariance matrices of two distributions.

## 3. Experiments

In the experiments, the following default parameter settings were used ($T_{d}=24$ h, δ=15 min, $\sigma^{2}=10$, N=5, B=256). The hyperparameters and structure of the proposed SSDM network were used in the experiments, as shown in Table 1. The *B* represents batch size and the learning rate of the neural networks was set to 0.01. The *Q* represents the number of features. The optimizer uses the Adam optimizer. Resnet18, and CNN adopt the same initialization strategy, optimizer, and learning rate decay method as SSDM to ensure the comparability of results.

Traditional machine learning method support vector machine (SVM) algorithms were also compared. The SVM algorithm constructs an optimal hyperplane to maximize the inter-class margin through constrained optimization, thereby enhancing generalization capability [19]. Specifically, the temporal sensor data sequences were directly employed as input features for hyperplane training to ensure experimental consistency. A Gaussian radial basis function (RBF) kernel was selected for nonlinear mapping, with its bandwidth and regularization parameter being jointly optimized via grid search across logarithmic scales.

### 3.1. Case of HLR

**Figure 4:**
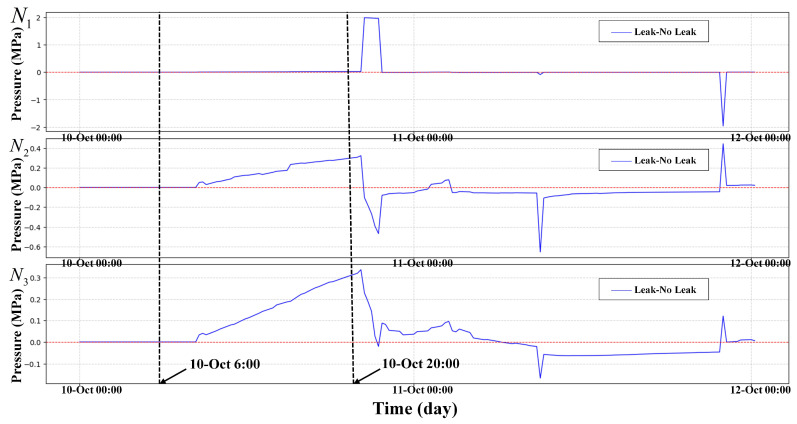
Leakage data minus normal data of three nodes $N_{1}$, $N_{2}$ and $N_{3}$.

**Figure 5:**
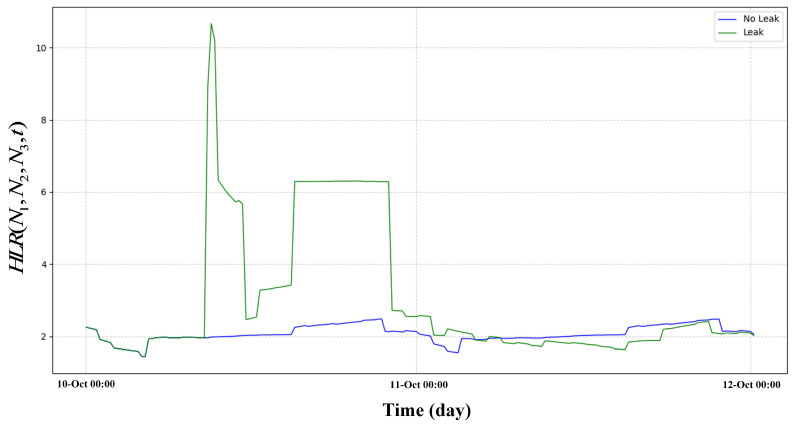
Leakage and non-leakage status of HLR.

To prove the effectiveness of HLR. A leakage incident of σ=0.1 was simulated in the EPANET sample network net3, where σ represents the proportion of the leakage orifice to pipe diameter. Set the sensor sampling interval to 15 min and the sampling duration to 48 h. The leaked node IDs are 129, 101, 240, 311, 125. Five nodes set a leak of size σ=0.1 at the same time. There is no leak at the beginning time of the simulation. Leaks happened at 6:00–20:00, on 10th October 2024. And, data from three pressure monitoring points named $N_{1}$, $N_{2}$, and $N_{3}$ were selected for analysis. $N_{1}$, $N_{2}$ and $N_{3}$ correspond to nodes 50, 269, and 249, respectively. The node requirements are set by default in net3. In Figure 4, we show the pressure differences of three nodes in both leakage and non-leakage states. The HLR curves in both leakage and non-leakage states are shown in Figure 5.

In Figure 4, it can be seen that the waveforms of the three nodes $N_{1}$, $N_{2}$ and $N_{3}$ all have small and irregular fluctuations when leakage occurs, but the fluctuations are small and the fluctuation time is short. Moreover, it is easy to confuse the pressure fluctuation caused by the change in the user’s water consumption habits with the pressure fluctuation caused by leakage.

We use the original leaked data of $N_{1}$, $N_{2}$, and $N_{3}$ to compute its HLR and compute the HLR of the non-leaked data of nodes $N_{1}$, $N_{2}$, and $N_{3}$ and plotted in Figure 5. In Figure 5, we can observe that the HLR exhibit significant differences between the leaked state and the non-leaked state. This has been well demonstrated in the observation results. In contrast, we can observe in Figure 4 that the pressure itself only has a small pressure drop. In contrast, pressure itself only has a small pressure drop in many leaks. This makes it difficult for us to detect such small pressure drops. This proves the importance of introducing HLR.

**Figure 6:**
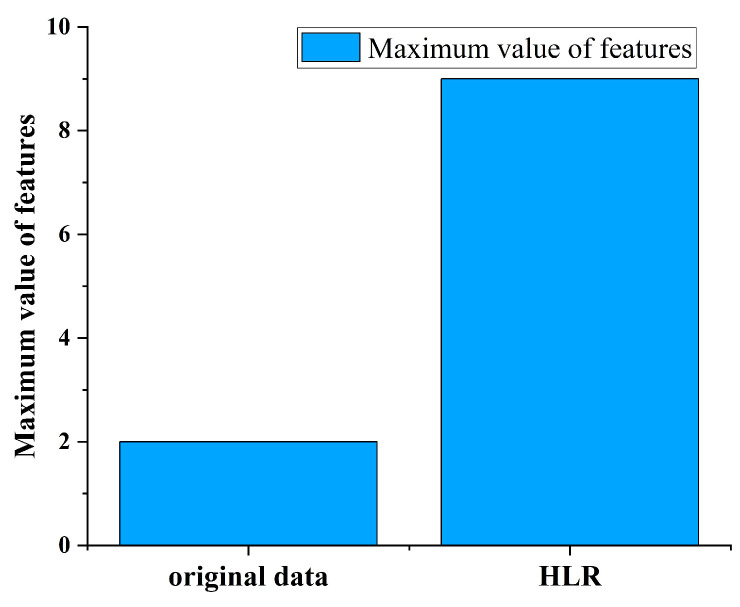
Comparison of the original data leakage features and HLR leakage features.

Figure 6 shows a plot of the maximum pressure drop difference in node $N_{1}$, $N_{2}$, and $N_{3}$ compared to the maximum difference for $HLR(N_{1},N_{2},N_{3},t)$. It can be seen that HLR have greatly improved the characteristics of data, with a 350% increase in leakage features.

### 3.2. Case 1

**Figure 7:**
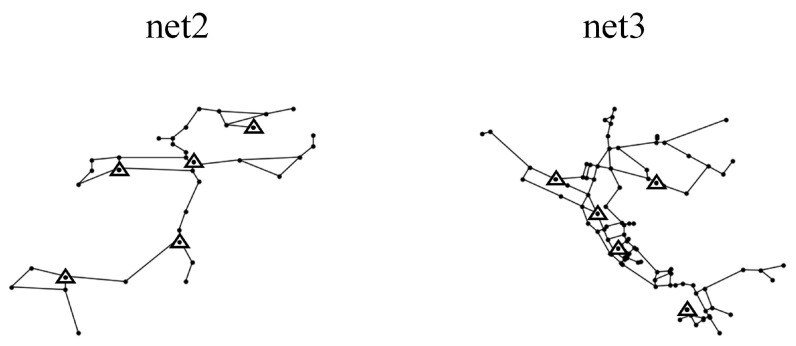
The placement result of sensors and layout of net2 and net3.

Two WDNs (net2, net3) were studied to validate the validity and reliability of the proposed method. We only used the pressure data of nodes as input for the model. net2 and net3 were two examples of standard pipe networks provided by the US EPA [40]. The topologies are shown in Figure 7. Among them, the net2 consisted of 25 nodes, 40 pipes, 1 water tank, and 1 reservoir. The pipe diameters were 200 mm to 400 mm and the node demands are 0 to 34.78 L/s. The different nodes had different node demands. The net3 consisted of 92 nodes, 117 pipes, 3 water tanks, and 2 reservoirs. The diameters of the pipes ranged from 100 mm to 500 mm. The node demands were 0 to 231.4 L/s. And, the different nodes were assigned different day and night demand patterns.

Before training, we first measure the similarity between the source domain and the target domain to prevent the negative transfer caused by an excessive distribution distance. The distribution distance between the source domain and the target domain is calculated to measure the degree of difference and ensure the effectiveness and necessity of migration. The distribution distance between net2 and net3 is shown in Table 2:

#### 3.2.1. Placement of Pressure Monitors

According to the K-means algorithm introduced in Section 2.4, we adjusted the node demand and record the water pressure changes at different nodes, respectively, to obtain the pressure leakage sensitivity matrices for net2 and net3, cluster the row vectors of the pressure leakage sensitivity matrices, and select the cluster centers as the pressure monitoring points. The placement results of the pressure monitoring in net2 and net3 are shown in Figure 7. Since the knowledge transfer was performed between different WDNs, Case 1 therefore carried out a binary classification task to diagnose the leaks in the pipes.

Used net3 as the source domain and net2 as the target domain. We simulated 3000 source domain data and 1000 target domain data under different operating conditions using the method described in Section 2.2 [41].

#### 3.2.2. Compared in Different Conditions

The proposed SSDM model was compared under different conditions to verify its validity:

1. Compared to the prediction accuracy of the model in different burst sizes ($\gamma_{\min},\gamma_{\max}$), the effectiveness of the SSDM model for small leak detection was observed;
2. Compared to the prediction accuracy of the model in different target domains with different numbers of samples, the robustness of SSDM under fewer samples conditions was observed;
3. Compared to the prediction accuracies of the models in different durations $T_{d}$, a longer duration may mean higher detection accuracy. But, it also reduced the real-time performance of the system detection. Therefore, it was necessary to find a suitable duration to improve the real-time and stability of the system;
4. The prediction accuracies of the models in different training batches are compared. Training batches with a good model fit are identified.

#### 3.2.3. Results and Discussion

To test the detection performance of our model, the source domain data under all operating conditions and 60% of the target domain data were put into SSDM for training. For CNN, Resnet18, and SVM, training does not distinguish between the source domain and the target domain. After training, 40% of the data in the target domain are extracted as test data. All models perform binary classification tasks.

From Table 3, it can be seen that our method achieved the highest detection accuracy, while the traditional machine learning method SVM had a lower detection accuracy of only 76%, so it is not used for subsequent comparison. This is our method, which, due to the introduction of HLRs, makes many small leakage features more prominent and can detect many small leaks. However, due to the introduction of HLRs, changes in user water usage habits and changes in water pressure caused by the use of fire hydrants were also detected as leaks, resulting in a false alarm rate that is 0.55% higher than Resnet18.

To verify the setting in Section 3.2.2, we extracted a specific number and leakage size of data from the target domain and trained and tested SSDM separately.

**Figure 8:**
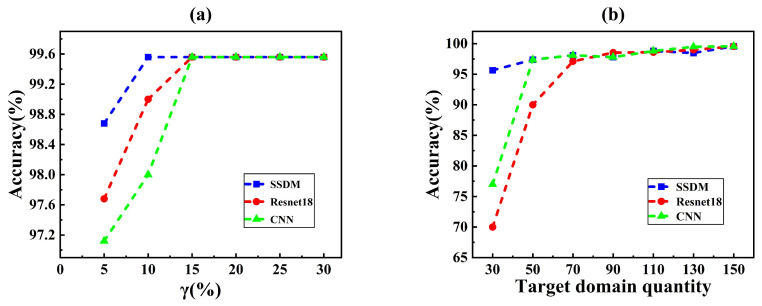
Accuracy comparison of the three methods for (**a**) different burst flow range and (**b**) different number.

As shown in Figure 8, the accuracies of the different methods were compared using net3 as the source domain and net2 as the target domain. Figure 8a shows the accuracy in different burst flow range. The accuracy increased when corresponding to a burst flow rate of 22.9 L/S for a 200 mm pipe at 30 m pressure with a leak diameter of about 0.0429 m (=10%), for which the SSDM accuracy was 98.68%, which was about 2% higher than that of the conventional neural network. Because HLR was introduced in the proposed SSDM, which amplified the pressure fluctuation of the small burst and enabled the neural network to extract more obvious features, this resulted in improving the detection accuracy. When raised to 15%, the accuracy of all three methods increased to 99.56%. The higher the burst flow rate, the more obvious the pressure perturbation was, which is more helpful for pattern recognition and leak diagnosis. In conclusion, the SSDM can achieve high accuracy under small leakage conditions.

Figure 8b shows the result of different numbers of samples. When the number of samples in the target domain was 30, the accuracy of SSDM was 95.56%, the accuracy of Resnet18 was 70%, and the accuracy of 1DCNN was 77%. The SSDM is about 20% more accurate than traditional neural networks under the small sample, which demonstrates the effectiveness of the proposed SSDM under small-sample condition. This was made by the introduction of transfer learning. Transfer learning allows neural networks to learn the potential features of different leaks from the source and target domains. The neural network cannot learn the features of different leaks due to a small amount of leakage data. Therefore, SSDM effectively solves the problem of low accuracy due to small amount of data.

Based on the above analysis, the proposed SSDM can obtain excellent results in all working conditions. Especially in the few-sample condition, where it outperforms Resnet18 and CNN by almost 20%. The SSDM can learn discriminative features from a small amount of data when the target domain data are limited. Thus, a competitive model can be obtained by training the model with only a small amount of target domain data. Overall, the SSDM could achieve higher accuracy than traditional neural networks under both the few-sample conditions and the small-leakage conditions.

**Figure 9:**
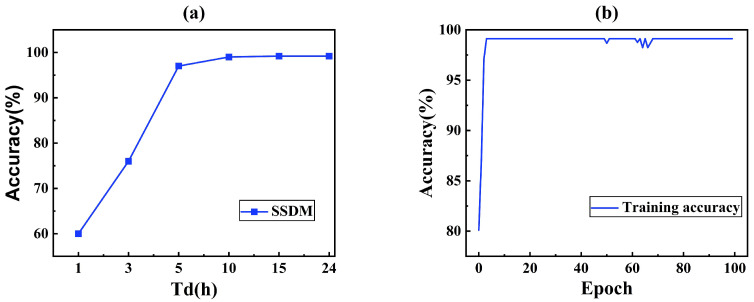
Accuracy of SSDM with (**a**) different data collection duration and (**b**) training epoch.

Figure 9 shows the effect of data collection duration and training epoch. It was expected that the longer the duration of data collection and the larger the training batch, the better the learning effect. The training epoch indicated the number of times that SSDM repeats learning the same training dataset.

As shown in Figure 9a, the accuracy was very low in the case of less than 3 h. Due to the fact that some of the leaked data had a starting leakage time greater than 3 h, and thus, the model was unable to learn the features of the leaked data, resulting in a very low accuracy. If the leakage time was 5 h, the accuracy of SSDM reached 97%, and the accuracy increased with the increase in the leakage time. And, if the leakage time reached 6 h, the growth rate becomes very slow and stabilized at 99%. Therefore, in order to ensure high accuracy, it was desirable that the length of the data was not less than 5 h.

Figure 9b shows that the SSDM reached convergence at 80 training batches (Accuracy > 99%). In this case, training each batch takes about 10 s on a computer with i5-9300H CPU, GTX1650 GPU. Thus, the SSDM could be trained well in a short time.

**Figure 10:**
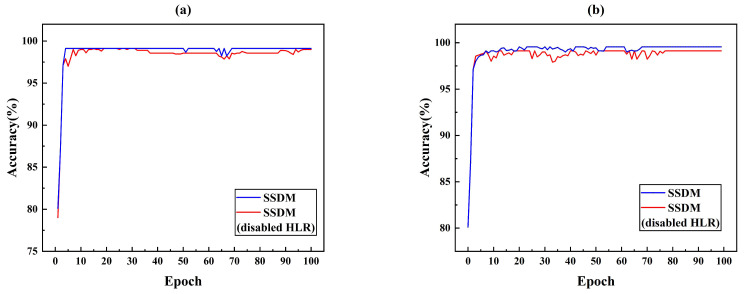
Ablation experiments with HLR (**a**) case of small leakage and (**b**) case of small simple.

The proposed SSDM method was subjected to ablation experiments with SSDM and SSDM (disabled HLR). Figure 10a simulated the accuracy of SSDM and SSDM (disabled HLR) in the case of small leakage. The accuracy of SSDM reached convergence in 80 epochs and achieves 99.12% detection accuracy. The SSDM method with disabled HLR reaches convergence at nearly 100 epochs and has 0.5% lower accuracy than the proposed SSDM method. Eighty samples are used as target domain data in Figure 10b. A comparison of the accuracy of SSDM and SSDM (disabled HLR) with fewer samples is simulated. The SSDM reached convergence at 69 epochs with 99.41% accuracy. The SSDM (disabled HLR) reached convergence at 77 epochs with 98.56% accuracy. The comparative analyzed in Figure 10 shown that the introduction of HLR improved the detection accuracy and convergence speed of the model. Because HLR could improve the features of small leakage and increase the feature dimension of input data, the model obtained more useful information, which improved the performance of the model.

### 3.3. Case 2

**Figure 11:**
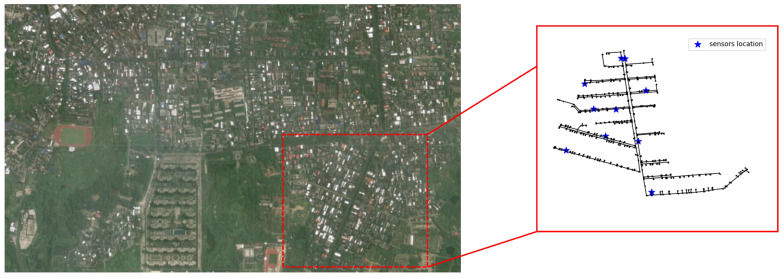
A real WDN in China and the structure of one DMA.

A real network and real data were used to test the reliability of SSDM under realistic settings. The network is located in the south of China, in Hainan Province. We selected one of the DMA partitions for analysis. This DMA partition consists of 686 pipes and 688 nodes. According to the needs of the project, the methodology of Section 2.4 was used to install 10 pressure monitoring points in this DMA. And this DMA is equipped with a complete SCADA online monitoring system. The real data were obtained in the Danzhou water service platform. And, the pressure data is transmitted remotely from the pressure sensor to the PC. The pressure sensors were placed by the Guangdong water company. The topology of the network and the location of the pressure monitoring points are shown in Figure 11.

The distribution distance between the source domain and the target domain is calculated to measure the degree of difference and ensure the effectiveness and necessity of migration. The distribution distance between the source domain data and the target domain data is shown in Table 4.

#### 3.3.1. Data Collection

**Figure 12:**
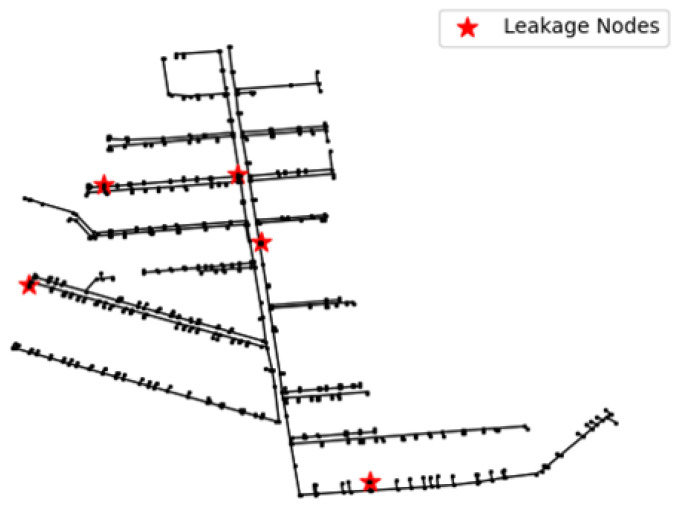
Specific locations of the 5 leaks.

To test the performance of the model in real-world applications, we conducted a total of five leakage simulations in mid-October–mid-November 2024 in this DMA partition. The time, weather, and content of their simulations are given in Table 5. The locations of the release points are labeled in Figure 12. We simulated pipe burst by attaching a drain valve externally to the pipeline. Small leakage means that the maximum pressure difference during the leakage period is less than 0.05 MPa, and large leakage means that the maximum pressure difference during the leakage period is more than 0.1 MPa. 0.05 MPa–0.1 MPa means medium leakage, and there was no medium leakage in the experiment.

The source domain data were generated by the method described in Section 2 using EPANET and the data were augmented. The duration of the simulation is 24 h and the sensor sampling interval is 15 min. We simulated net3 for 3000 different leak locations and different leak sizes. Then, a random perturbation of (−10, 10) and a Gaussian white noise with a mean of 0 and a variance of 1 were added to these 3000 data. The target domain data were the real data in this DMA. We cut it into time series of the same length as the source domain data and fed these into the network for training, which consisted of 5 leaked data and 10 non-leaked data.

#### 3.3.2. Result

The training strategy of Algorithm 1 was used. And, the classifier for this DMA partition was obtained by training on source domain data and target domain data.

Five real leakage data tests were performed on the collected data. The test results are shown in Table 6, where label 1 denotes leakage and label 0 denotes non-leakage.

**Figure 13:**
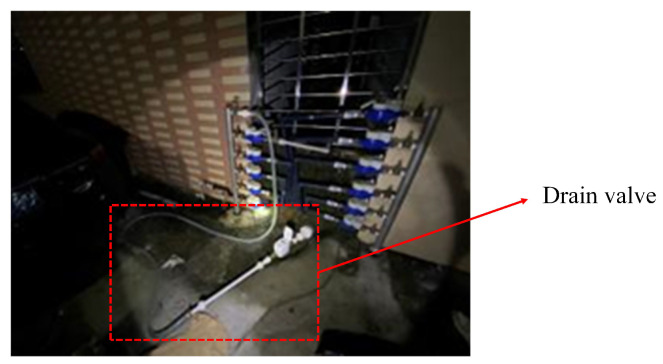
Working condition diagram of manufacturing leakage C at night.

It can be seen that four out of five leaks can be detected, even in the more remote location and smaller leak C, the leakage condition of leakage C is shown in Figure 13. This shows the effectiveness of the algorithm for monitoring small leaks.

**Figure 14:**
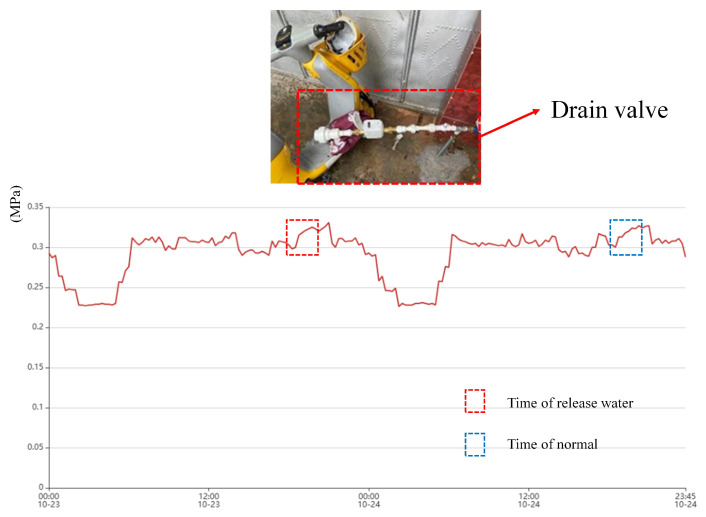
Working condition at the site of Leak A and daily water pressure time series.

Upon inspection, Leak A, which was misclassified as a non-leak, was due to the fact that the manufactured leak was too small and at a peak water usage time, and the location of the manufactured leak was too far away from the sensor. An examination of the data in the system showed that none of the sensors fluctuated significantly during this peak water use period. As shown in Figure 14. Thus, it is difficult to effectively detect small leaks that are generated for peak times.

**Figure 15:**
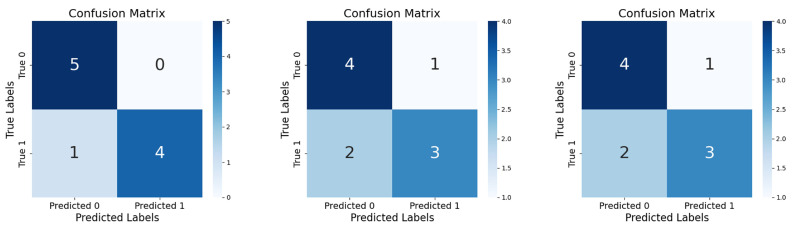
Confusion matrix for SSDM, Resnet18, CNN.

Finally, to verify the accuracy of our algorithm, we performed classification using five leakage data and five normal water data. Comparison was performed with a conventional CNN, Resnet18. The confusion matrix is obtained as shown in Figure 15.

It can be seen that both Resnet18 and CNN have two misses in the test, which illustrates the effectiveness of transfer learning and HLR. The proposed algorithm can realize the detection of leakage partitions by feeding the data of each partition into the trained classifier separately. By narrowing down the burst pipe to a certain range, the exploration time and workload are effectively shortened. The method provides a feasible idea for actual engineering practice.

## 4. Conclusions

In this paper, a novel deep transfer learning method (SSDM) was proposed to solve the problem of limited data in the burst diagnosis of WDNs in real industries. This method combines the principles of hydraulic physics with DTL to solve the key challenge of insufficient data in burst detection of real-world WDNs. By embedding HLR into the neural network, SSDM combines physical- driven and data-driven features through a layered fusion architecture, resulting in a 0.5% increase in detection accuracy for leaks. The introduction of transfer learning reduced the required target domain samples by 57%, while maintaining an accuracy of 95% in experiments with few target domain samples. Unlike methods that rely on high-frequency sensors, SSDM combined with low-frequency pressure sensors reduces hardware costs and can be deployed in municipal WDNs. This method demonstrates great potential for application. However, SSDM relies on the water pressure changes generated during leakage to detect leaks, which is limited for WDNs with extreme hydraulic changes. The use of SSDMs can be limited when a particular pressure sensor fails or when there are large consumers with frequently changing water needs. Moreover, SSDM can only be used for the regional inspection of large water supply networks, and the use of SSDM can be limited to water distribution systems with highly branched networks. In the future, more robust models should be obtained by combining flows or other forms of data to adapt to diverse WDN configurations. This may require more different types of sensors and more developed WDN systems, cost more money, and require the fusion analysis of multimodal data.

## Figures and Tables

**Table 1:** Parameters of network size in SSDM.

Module	Input Size	Output Size
Source feature module	[B, 97, Q]	[B, 512, 512]
Source bottleneck	[B, 512, 512]	[B, 128, 128]
Target feature module	[B, 97, Q]	[B, 512, 512]
Target bottleneck	[B, 512, 512]	[B, 128, 128]

**Table 2:** The deviation degree of source data and target data.

Type	Distance
MMD	1.5604
CORAL	0.1977

**Table 3:** Accuracy, recall, and false alarm rate (FAR) of four methods.

Model	Accuracy	Recall	FAR (%)
SSDM	99.41	99	1.8
Resnet18	99.15	98.5	1.25
CNN	98.89	99	1.34
SVM	76	81	9.4

**Table 4:** The deviation degree of source data and target data.

Type	Distance
MMD	1.6811
CORAL	0.2523

**Table 5:** The station of 5 real leaks.

Number	Time	Weather	Users Water Consumption	Leak Size	Point
A	2024-10-23 Wednesday p.m.	Fine	Peak	Small	500
B	2024-11-4 Monday p.m.	Rain	Normal	Big	510
C	2024-11-6 Wednesday morning at dawn	Fine	Low	Small	600
D	2024-11-12 Tuesday p.m.	Fine	Normal	Big	558
E	2024-11-16 Saturday noon	Fine	Peak	Big	739

**Table 6:** Test results.

Number	Real Label	Test Label
A	1	0
B	1	1
C	1	1
D	1	1
E	1	1

## Data Availability

The dataset generated and analyzed during the current research period is divided into simulated data and real data. The hydraulic software analysis package that supports the generation of simulated data for this study is WNTR, which is a Python package compatible with EPANET designed to simulate and analyze the elasticity of water distribution networks. Can be in https://usepa.github.io/WNTR/index.html (accessed on 25 August 2024) Get it for free. Real data can be obtained from the corresponding author and licensed for free.

## Abbreviations

The following abbreviations are used in this manuscript:

WDNs	Water Distribution Networks
SSDM	Small Sample Diagnosis Method
HLR	Head Loss Ratio
CORAL	Correlation Alignment
MMD	Maximum Mean Discrepancy
EPANET	Environmental Protection Agency Network Analysis Tool
DMA	District Metered Area
DTL	Deep Transfer Learning
